# “Without IPS I Think I Would Really Fall Apart”: Individual Placement and Support as Experienced by People With Mental Illness—Phenomenological Peer Research Study

**DOI:** 10.1177/10497323241275046

**Published:** 2024-10-21

**Authors:** Marianna Agata Borowska, Kristin Berre Ørjasæter, Marit Borg, Barbara Stenvall, Alexandra Silbermann, Miles Rinaldi, Eóin Killackey, Arnstein Mykletun, Cathrine Moe

**Affiliations:** 1 Faculty of Nursing and Health Sciences, 1786 Nord University, Bodø, Norway; 2 Faculty of Nursing and Health Sciences, 1786 Nord University, Namsos, Norway; 3 Faculty of Health and Social Sciences, 177041 University of South-Eastern Norway, Drammen, Norway; 4 Centre for Work and Mental Health, Nordland Hospital Trust, Bodø, Norway; 5 South West London & St George’s Mental Health NHS Trust, London, UK; 6 Centre for Research and Education in Forensic Psychiatry, Haukeland University Hospital, Bergen, Norway; 7 188668 Orygen The National Centre of Exellence in Youth Mental Health, Parkville, VIC, Australia; 8 Centre for Youth Mental Health, The University of Melbourne, Melbourne, VIC, Australia; 9 Department of Community Medicine, UiT – The Arctic University of Norway, Tromsø, Norway; 10 Division for Health Sciences, Norwegian Institute of Public Health, Oslo, Norway

**Keywords:** individual placement and support, severe mental illness, vocational rehabilitation, peer research, everyday life, phenomenology, reflective lifeworld research

## Abstract

Having a job is an important component of recovery from mental illness and a source of economic, social, and health benefits. Most people experiencing severe mental illness (SMI) want to work but are excluded from employment opportunities. Employment specialists (ESs) working in individual placement and support (IPS) teams help persons struggling with SMI obtain competitive employment. This study is a qualitative phenomenological study of 10 IPS participants in the Norwegian context, serving to develop a deeper understanding of the IPS phenomenon as it is experienced in the everyday life of IPS participants. The study was designed as a peer research project including four members of a competence group with experience in IPS and SMI. The results, analyzed using the reflective lifeworld research approach, revealed four constituents: “Having a safety net along the way toward employment,” “Feeling more like a person, not just a patient,” “Brighter future,” and “Going above and beyond employment support.” IPS functions as an anchor in participants’ journey toward employment. Strong and meaningful relationships with an ES seem crucial for IPS participants to gain the strength and confidence essential to engage in the job search. IPS participants experience various challenges in everyday life, resulting in ESs exceeding their vocational role to cover the unmet needs that health services seem unable to fully address. Closer coordination between vocational and health services, as well as a deeper focus on everyday life issues, will positively affect IPS participants’ vocational outcomes and quality of life.

## Background

Everyone has the right to work (United Nations, 1948, art. 23), and work is a central conduit to justice for the disability rights movement (Blattner, 2021). Mental illnesses, with their high prevalence and impact, are the major cause of disability in Western countries, and the disability rate is growing (Goldblatt et al., 2023; World Health Organization, 2022). Employment is an important context for recovery from mental health problems (Borg & Kristiansen, 2008; Drake & Whitley, 2014), a source of economic and social benefits (Burton & Waddell, 2006; Modini et al., 2016), and a critical health intervention (Knapp & Wong, 2020). Most people experiencing severe mental illness (SMI) want to work, but few succeed in obtaining and retaining a job (Modini et al., 2016).

Traditionally, people experiencing SMI were thought to be unable to work (Warner, 2005). Recently, there has been growing interest in the relationship between work and mental health (Henderson et al., 2011), particularly regarding workplace risk factors for mental health (Harvey et al., 2014). Contrary to expectations, increasing evidence suggests that symptom remission is not required to be able to work (Provencher & Keyes, 2011; Russinova et al., 2005). Furthermore, competitive work can be beneficial for those experiencing active SMI symptoms and medication side effects and might even decrease symptoms of mental illness (Anthony, 2008; Marrone & Golowka, 1999). However, people experiencing SMI are exposed to stigmatization in the workplace (Glozier, 1998; Hampson et al., 2020), and the experience of stigma is still present when successfully employed in regular jobs (White et al., 2023).

### Individual Placement and Support

Individual placement and support (IPS) is an evidence-based practice of vocational rehabilitation that aims to help people experiencing moderate to SMI obtain and maintain competitive employment in ordinary jobs (Bond & Drake, 2014). Despite being the most effective vocational rehabilitation model (Brinchmann et al., 2020), only around 44% of those using IPS in Europe manage to retain their jobs over time (Drake et al., 2019), and most jobs are part-time (Corbière et al., 2020), leaving room for further development. The IPS model is founded on eight evidence-based principles: focus on competitive employment, eligibility based on client choice, integration of mental health and employment services, attention to client preferences, individualized job-finding assistance, rapid job search, systematic job development, and time-unlimited individualized support (Bond & Drake, 2014).

IPS teams typically include employment specialists (ESs), employment and benefit advisors, and health service professionals (Moe, Brinchmann, et al., 2023; Moe, Hansen, et al., 2023). IPS is intended to be integrated into clinical mental health treatment (Bond & Drake, 2014). The key role of an ES is to work closely with IPS participants to support them in finding and retaining a competitive job of their choice. ESs intend to focus exclusively on vocational tasks and coordinating vocational plans with other team members by using the IPS approach (Moe et al., 2021; Rinaldi et al., 2008); they are not intended to be care coordinators (Rinaldi et al., 2008).

Most research on IPS has been quantitative (Moen et al., 2021; Rahbæk Møller & Bonfils, 2024), focusing mainly on effectiveness (de Winter et al., 2022). This can be at the expense of the experiences, values, and processes impossible to express in terms of numbers and percentages (Nygren et al., 2016). Traditionally, little attention has been paid to the experiences of people experiencing SMI, their perceptions of the IPS model compared to traditional vocational rehabilitation programs, or their experiences of the job search and return to work (Coombes et al., 2016; Koletsi et al., 2009). However, lately, an increasing number of qualitative studies have focused on participants’ experiences with IPS. The results indicate the importance of including personal experiences to improve IPS implementation and employment outcomes (Dawson et al., 2021; Kwan et al., 2021; Vukadin et al., 2021).

IPS has been evaluated positively by participants, with the approach promoting person-centered and recovery-oriented care (Dawson et al., 2021). Practical assistance in the job search process and improving work-related skills and knowledge, as well as personal support in the form of encouragement in building self-confidence, have been highlighted (Boycott et al., 2015; Johnson et al., 2009; Liu et al., 2007).

Qualitative studies demonstrate the significance of a collaborative relationship between ESs and the IPS participants in which participants experience a feeling of control (Areberg et al., 2013). While the relationship with ESs is seen as fundamental to the engagement of IPS participants in the job search process (Kinn et al., 2021; Rahbæk Møller et al., 2020), some studies involving ES competencies explore the links between working alliance and job acquisition. Catty et al. (2008) and Corbière et al. (2024) identified that a better working alliance between the ES and IPS participants predicts better chances of acquiring a competitive job, whereas Kukla and Bond (2009) did not identify such an association. This indicates that relational aspects between ESs and IPS participants remain inadequately explored.

More qualitative results need to be incorporated in IPS evaluations and developments to understand how well IPS engages potential service users (Park et al., 2022). The existing qualitative research on experiences with the IPS model is limited to how the relationship with an ES influences IPS participants on the individual level, not discussing organizational and societal contexts of IPS or IPS in relation to health and social services (Rahbæk Møller & Bonfils, 2024; Rahbæk Møller et al., 2020). Actions on the microsystem level are integrated and connected to broader contextual, environmental, and societal mechanisms and factors (Homa & DeLambo, 2015). This study addresses the gap in the literature and explores the relationship between ES and IPS participants in the societal and organizational context, unveiling how IPS works in practical settings, particularly in relation to welfare services. In addition, people with lived experiences are not typically included in conducting research on IPS (Rahbæk Møller et al., 2020). This study utilizes peer research method by including the voices of persons with lived experiences in all stages of the research process. The purpose of drawing on their experiential knowledge is to foster democratic development of knowledge on IPS and strengthen study’s utility and relevance for practice (Ives et al., 2013).

Overall, the aim of this study is to develop a deeper understanding of the IPS phenomenon as experienced by IPS participants in everyday life.

## Methods

### Design

We employed a peer research design, wherein people with relevant lived experiences take part in directing and conducting research (Lushey, 2017). For many years, people with lived experiences have been advocating for their greater involvement in the production of knowledge in mental healthcare (Rose, 2004; Sweeney, 2013). They have demonstrated a need for more contextually oriented everyday life research based on their own experiences, needs, and interests, focusing on living conditions, money, employment, education, stigmatization, and social exclusion (Borg & Askheim, 2010). Consequently, there has been a considerable expansion of interest in and the practice of involving persons with lived experiences in research and challenging the basis of mental health knowledge (Faulkner, 2017). This has resulted in established and ongoing knowledge production rooted in the collective experience of people who have used mental health services (Rose et al., 2018). Thus, a competence group was established for this study, consisting of four persons with lived experiences with mental illness and IPS—two women and two men—representing different age groups. This group was involved in developing the content, language, and structure of the interview guide. The competence group’s language expertise resulted in creating questions that would not be experienced as patronizing or stigmatizing (Devotta et al., 2016). The competence group also participated in data collection, analyzed data together with the first author (MB), translated from Norwegian to English some parts of the analyzed data, and took part in disseminating the results. A course in collaborative research was organized in preparation for the study (Moe et al., 2022).

### Reflective Lifeworld Research

To obtain a deeper understanding of the IPS phenomenon as experienced in everyday life, descriptive reflective lifeworld research (RLR) was used. RLR is rooted in phenomenology traditions that provide a non-reductionist, non-dualistic, and holistic view on human existence (van Wijngaarden et al., 2017). The RLR focus is on how the world and its everyday phenomena are acted, lived, experienced, and described by humans (Dahlberg et al., 2008). The lifeworld is a core concept in phenomenology, understood as the world we as human beings live in, the pre-theoretical world of experience that we typically do not question (Zahavi, 2018). The lifeworld is the foundation of understanding humans, life, health, suffering, and well-being (Johansson et al., 2009).

The research process was guided by an attitude of openness. The idea of “bridling” covers “bracketing” and is understood as refraining from pre-understanding, as personal beliefs, theories, and other assumptions that can limit openness. Bridling also covers refraining from understanding too quickly and not making definite what is indefinite. This opens up the possibility for the phenomenon to present itself in order to explore possible meanings and understandings (Dahlberg & Dahlberg, 2020). The “bridled attitude” was applied as a methodological approach throughout the research process. All researchers consistently reflected on their understanding of the phenomenon, challenging their own pre-understanding and refraining from understanding too quickly. Several critical discussions on the reflective process were held.

### Study Context

Based on positive results in other countries, the Ministry of Labour and Social Inclusion (2016) and the Norwegian Directorate of Health (2016), together with the Labour and Welfare Administration (NAV), established systematic collaborations in implementing the IPS model. National guidelines for the diagnosis, treatment, and follow-up of persons experiencing psychotic disorders recommend the IPS model as a favorable approach in the job search process (Norwegian Ministry of Health, 2013). Norwegian health and welfare services are rooted in different sectors, funded separately, and regulated through different legislation (Moe, Brinchmann, et al., 2023). The IPSNOR project was established in reaction to difficulties experienced in IPS implementation and aims to scale up IPS in Northern Norway (Moe, Hansen, et al., 2023). Our study is an independent study but at the same time is part of the IPSNOR project.

Norway’s latest National Action Plan for mental health concluded that work inclusion of persons struggling with SMI has been underprioritized and should be incorporated into treatment. Further implementation of IPS is recommended as a part of cooperation between the Norwegian Labour and Welfare Administration and health services (Ministry of Health and Care Services, 2023).

### Recruitment and Participants

Ten persons—six women and four men aged 25–54 years—were recruited for interviews. Participants were currently in treatment at a mental health service that offers IPS in Northern Norway. Following the ontological and epistemological principles of lifeworld research, meanings are infinite, and therefore the issue of meaning saturation was not raised (Dahlberg et al., 2008). Sample size was guided by the principle of variation (Dahlberg et al., 2008) and included informants of different ages, genders, and experiences with IPS and SMI to ensure rich variation in the data. Purposive sampling (Etikan et al., 2016) was employed, ensuring that the chosen services were in accordance with IPS principles and met the inclusion criteria for the study: (a) in regular contact with ES based on experiencing moderate to SMI and (b) receiving support from mental health services. The participants were recruited through three ESs belonging to two IPS services in Northern Norway, who provided oral and written details about the aim, procedures, possibility of withdrawal, and potentially negative and positive effects of participation in the study. Participants who decided to take part in the study contacted the researcher by telephone, who again explained details of the study and answered questions from the participants. Appointments for the first interviews were scheduled. Before interviews started, participants in the face-to-face and telephonic/Microsoft Teams interviews provided written or oral informed consent, respectively.

### Data Collection

Qualitative phenomenological research was conducted, and rich data regarding the studied phenomenon in the everyday context were collected (Dahlberg et al., 2008). Data were obtained through in-depth interviews with participants from January 2020 to June 2022. After conducting 10 interviews in Norwegian: four face-to-face, one telephone, and five over Microsoft Teams, we decided that we obtained the variation of the data and stopped recruitment for the study. After approximately 2 months, we conducted eight follow-up interviews to develop a more detailed understanding of the phenomenon, including the possible new aspects illuminated through the research process. Interviews lasted 1–1.5 hr and were voice recorded. Three different data approaches to collecting data were caused by COVID-19 pandemic restrictions that made conducting solely face-to-face interviews challenging. We noticed that face-to-face interviews lasted around 30 min longer than telephone and Microsoft Teams interviews and provided richer and more detailed data. It is difficult to conclude if this was caused by a potentially safer and more intimate character of meeting in person with participants or if there were other factors that could influence the interview situation. Interviews were led by MB, together with one representative from the competence group. A competence group member was present during interviews, based on the concept of expertise by experience, reflecting the idea that identity produces knowledge (Gallacher & Gallagher, 2008) and that the competence group could obtain information not fully accessible to MB as an outsider (Devotta et al., 2016; Nind, 2014). Competence group members had an active role during interviews, building an environment of trust and helping guide the participants through the questions. Guided by their experiential knowledge, competence group members were responsive to the participants’ answers and able to ask additional relevant questions to increase the understanding of the studied phenomenon.

At the time the first interviews were conducted, seven participants were in the job search process, one had a full-time job, one had a part-time job, and one was a casual employee. Participants’ employment situation remained unchanged at the second interview. During the interviews, participants were asked to tell the researchers about their experiences with IPS, encouraging them to give rich descriptions of this phenomenon in the everyday life context. Witnessing the contextual variations in everyday life and emerging variations of meanings in their experiences helped see the structure of the phenomenon (Dahlberg et al., 2008). After each interview, reflection notes were made followed by a critical discussion between the competence group member present and MB.

Interviews were transcribed verbatim by MB. Following Kvale (1996), MB transcribed all material personally to ensure that the context, richness of meanings, and language accuracy were preserved.

### Data Analysis

Descriptive analysis in line with the RLR was conducted. MB and the competence group worked together with the lifeworld descriptions of participants’ experiences, aiming to understand the IPS phenomenon without interpretation, explanation, or construction (Dahlberg et al., 2008). The interviews were first read by MB several times, moving between the whole data material and in parts. The bridled attitude was applied to be as open and reflective as possible. First, units of meanings related to each other and to the text as a whole were temporarily put together in the larger patterns of meaning clusters. These clusters were presented to the competence group, and all researchers worked collaboratively to look for both particular and essential meanings of the phenomenon. Meanings of IPS as experienced and lived by participants in everyday life showed themselves in the process of understanding intentionality in participants’ descriptions of the phenomenon. To get closer to the structure of meaning, the following questions were asked: “What does this participant voice and what does this mean?” “Why is this how we understand this?” and “Is there another way to understand this?” Critical discussion and including perspectives of people with lived experiences were important to increase the credibility of the analysis. Constituents—the meanings that make up the actual essence—were identified reflectively and inductively. The research process led to revealing a new written understanding of the phenomenon being studied. Data were analyzed in Norwegian in order to grasp the meanings of the phenomenon as exact and precise as possible. Later, one member of the competence group translated quotes from the data used in the Results section into English. The translation was then carefully considered by the competence group and MB to secure preserving of the precision and nuances of the original data.

### Ethical Considerations

The Norwegian Agency for Shared Services in Education and Research (Sikt) approved the study (reference number: 201844). Although approval was also sought from the Regional Committees for Medical and Health Research Ethics (REK), they concluded that the project fell outside their mandate (reference number: 184618).

Ethical principles regarding participants were taken under consideration owing to particular interview situations or personal characteristics that could make them vulnerable (Gordon, 2020). All participants were interviewed in a safe environment in the presence of a member of the competence group, with whom they might have felt more comfortable (Kristiansen et al., 2009).

Important ethical issues regarding conducting peer research were also raised several times. MB ensured that the role of the competence group was not reduced to symbolic participation. Instead, a common ground with those with lived experiences was established, and the competence group was involved in all stages of the research process. Both emotional support (peer support, debriefing) and research-related support (supervision) were offered. An open approach to everyone involved in the study and transparency about the theoretical aspect was ensured (Faulkner, 2004). Practical financial support from Nord University was granted to cover payments for the meetings with the competence group.

## Results

The new understanding of IPS, as experienced and described in everyday life by participants, is presented as the description of an essence. True to the RLR methodology, the essence of the phenomenon is introduced first, followed by descriptions of four constituents. The essence is articulated in the present tense, as it encapsulates the phenomenon’s current state, not the participants’ statements about it (Dahlberg et al., 2008). The meaning constituents exhibit some overlap, and the essence is inherent in each constituent. Each constituent is thoroughly explored and encompasses both the general meanings as well as the variations of meanings and unique experiences, supplemented with interview quotations.

### Essential Structure of Meaning

The essential meaning of the IPS phenomenon can be understood as an anchor in participants’ lives that prevents them from “falling apart.” The ESs, core actors in IPS, provide the continuity and flexibility essential to constructing a safe and secure environment for participants, thereby earning their trust. This support enables participants to embrace life changes and engage in job-seeking. By establishing a robust relationship with the ES, one that is rooted in respect, being heard and valued, and not being reduced to their SMI diagnosis, participants find empowerment to enhance their self-worth, essential to the process of advancing their vocational goals. Participants are perceived as equal human beings, endowed with interests and talents, and capable of living a fulfilling life as integrated members of society. The relationship between the ES and participants forms a steady foundation that is crucial to the participants’ personal growth. Feeling safe and cared for, coupled with improved self-esteem, empowers participants to foster hope that positive changes are attainable and eventually believe in a brighter future. When safety, higher self-esteem, and faith are established, opportunities open for engagement in both vocational and non-vocational activities. IPS, when applied in practical settings, transcends the scope of a vocational rehabilitation model; it embodies a holistic approach, addressing a broad range of individual needs. Participants, as beneficiaries of the welfare system, often face insufficient support from social services and inadequate follow-up from mental health treatment teams. They grapple with various challenges and voice the need for support in coping with daily activities. ESs, in this study, appear to exceed their vocational role and address these other needs, providing the type of support that meets individual expectations.

#### Having a Safety Net Along the Way Toward Employment

In the journey toward employment, having a safety net emerges as vital for participants. This safety net represents a mechanism that catches one, providing the necessary assistance to begin their employment journey. Integral to this safety network is the role of a trusted ally, a confidant with whom one can trustfully discuss not only the practicalities of job-seeking but also the broader life circumstances that shape their employment trajectory. This ally emerges as someone who is a beacon of genuine concern and goodwill, championing their success. ESs form a continuous support system, actively present from the outset, engaging participants in their job search and affirming to them the value of their endeavors. This support also extends to catch them in case of setbacks. Such assurance alleviates anxiety among participants, instilling resilience in participants to face the challenges. One participant encapsulates the profound personal nature of this security, stating, “The first step is to create the personal safety that makes you really feel safe in everyday life, that you can move forward” (P1). Having someone close by when needed serves a comforting sense of security: “It [IPS] gives me safety in a way, you can call it a safety wheel of support; of course, I still do my job myself, but they guide me and help me along the way, as it were” (P10).

ESs provide comprehensive assistance to IPS participants on their employment journey. This encompasses aiding them in finding jobs that resonate with their personal preferences and supporting them through the trials of securing and maintaining employment. P9 refers IPS as “A back-up in the working life,” reflecting the participants’ perception of ESs as a steadfast ally and personal contact, consistently available and approachable whenever needed. The ES’s flexibility and availability, including outside of standard working hours, are pivotal in fostering trust, which is fundamental to building a robust relationship: “The trust built up in time … I could send her a message around the clock if there was something. That made me trust her more” (P9).

ESs actively provide participants with a sense of alliance, demonstrating genuine care for their well-being and actively supporting their employment success. The ES role is not merely transactional; it is also affirming the participants’ worth beyond mere employment outcomes. This nurturing approach cultivates a sense of security and balance, allowing participants to feel in control of the process and confident that their boundaries are respected. A participant’s reflection illustrates this experience: “Having someone who can support you, there’s extra safety in it when there’s someone there who wants you to do well and wants you to work. Or not to work, if that’s the best thing for you right now” (P4).

The profound impact of the support system creates an environment that is not only secure but also empowering for participants, providing strength to explore possibilities and fostering personal growth:

It feels very safe to be able to explore things and, for the first time, talk about what I really wanted to work on. Being able to have a safe framework and then actually explore possibilities at work and outside of my comfort zone. (P7)

#### Feeling More Like a Person, Not Just a Patient

Struggling with mental illness and facing exclusion from work opportunities diminishes participants’ self-confidence and sense of agency. Participants share how the inability to engage in regular work activities deprives them of the sense of worth. Being treated as a whole person rather than defined solely by one’s mental illness shows the transformative potential of being seen, heard, and valued. Having someone who believes in participants, who motivates them and strengthens their abilities, makes it possible to envision and work toward a future that encompasses meaningful employment and personal fulfillment.

Joining IPS opens the door to forging a robust and meaningful relationship with an ES. Most participants recount experiencing being invisible and unheard within the mental health services, leaving them feeling devaluated and distrustful. In contrast, the bond with an ES represents a new chapter where they feel acknowledged and respected:

I often felt that those people [mental health professionals] sitting around in their offices don’t have ears. They just write some nonsense and get paid. They tell you that they will help, but they don’t. This is why I was very skeptical about ES’ in the beginning, but it did not last long. It was a person I met, not a robot, somebody that could understand. She heard me speak and she heard what I said! (P3)

Participants experience navigating a profound disconnection with mental health services, which appear to dwell on diagnosing and treating conditions, neglecting the nurturing path to recovery. The emphasis on “fixing” rather than healing leaves participants longing for a more supportive approach that acknowledges their struggles and guides them toward regaining their sense of self beyond their mental health problems: “There was a lot of talk about illness and not so much talk about what to do to make me feel better” (P4). Participants mention the significance of ESs recognizing their complete humanity, not just their ailments: “She [ES] is very curious about me, and she sees those healthy parts of me. She is not just seeking those healthy parts; she is able to find them, accept them, and relate to them” (P6). ESs transcend diagnostic labels, acknowledging participants as persons, guiding them to unearth and amplify their innate abilities. A participant recounts how an ES’s authentic engagement with her instilled a sense of value in her insight, bolstering her confidence and fostering growth:

[ES] very often asks … because I am very engaged in what I do; I love history and stuff and I just can go on and on about things I have learned at the university or to explain things that I am interested in and she may not have known about from before … This makes me feel that I am not totally useless, this gives me confidence that I need to pass my exams and to get through all this. I didn’t think I would be able to do this, so, yes … (P6)

The ability of ESs to demonstrate genuine interest in participants as valuable human beings clearly emerges from participants’ stories. This involves being acknowledged and listened to, treated like regular people with unique passions and capabilities, rather than merely as psychiatric patients. Such recognition forms the foundation of a stable, robust, and respectful connection with ESs. This active and empathetic approach cultivates a space where participants not only recover but also thrive. At the same time, ESs are actively supportive and eager in encouraging participants in their efforts. One participant shares, “I feel that I have a person who believes in me, and this keeps me going. It makes me feel stronger. It is not like this, yes, ok, you might try … No! She believes in me. ‘You’ve got this!’” (P2). These interactions significantly shape participants’ self-perception, boosting their self-esteem, and unveiling their strengths and resources: “I feel like a lot has changed. I haven’t got a job yet, but I have changed. For me it is personal growth that is most important. Without it I wouldn’t be able to keep a job” (P1).

#### Making the Future Look Brighter

In the absence of vocational opportunities and struggles with SMI, loneliness, and social exclusion, feelings of hopelessness pervade the everyday life of participants. This despair manifests in various forms, such as the attempts to disengage from social connections and the efforts to dismiss the burgeoning sensation of hopelessness: “Ok, now the thoughts about reality come back to me … No … I have become so awfully good at writing everything away, good at letting it all go away ….” (P1).

Active engagement in IPS and embracing the nurturing robust bond with ESs ignites hope and regains faith for the future. At the heart of this transformation lie two pivotal factors. First, an ES advocates for the possibilities, positive outcomes, and new beginnings within a safe environment rather than emphasizing barriers. One participant reflects on his path and the role of the ES guiding him through adversities, sharing a lived experience that resonates with moving beyond past failures.

I really didn’t want [ES] to dig up my past and analyze why it all fell apart. It was such a great feeling of failure, I just wanted to skim over it … And it went really well, we just went right past it, it didn’t really matter! And we could just go on, we could just move forward. (P1)

Second, the passionate involvement of ESs, their positive attitude in interactions with individuals with SMI, and the ongoing reassurance that employment possibilities were within reach fosters a belief that life still offers possibilities and that “Life wasn’t over yet” (P10). One participant shared his lived experiences of the impact of positive and encouraging attitude of the ES on his expectations about the future:

Before IPS, it was a little grey, a little dark kind of; I didn’t really believe that things could be okay, but then my IPS contact came along, like fireworks with positivity and joy, enthusiasm and glow, that mentality infected me basically. I gained hope that things actually could be okay in the end. (P7)

Being a part of IPS and having continuous support from ESs with the focus on possibilities helps to develop hope and strengthen belief in existence of satisfying vocational possibilities available for individuals struggling with SMI and that a change of positive transformation awaits.

#### Going Above and Beyond Employment Support

Participants illuminate the groundwork of the job search as a journey that extends beyond mere employment, revealing the necessity to address various life challenges before (or simultaneously) in order to being able to succeed in finding a job. Sometimes, the participants experience that there is a disparity between their own belief and the expectations of NAV when it comes to life management and job success:

The fact that (ES) herself thinks, “yeah, it’s good to have other things worked out first, before getting yourself a job.” Because that isn’t how NAV sees things. NAV doesn’t think you should fix the other areas in your life first; they think, “yeah now you have to do everything at the same time.” They don’t take into account that that can be very overwhelming, or really freaking impossible sometimes. (P3)

In the lifeworld of the participants, everyday challenges cast a long shadow, clouding their journey. Although being in mental health treatment, they still find themselves adrift, seeking supporters that could truly understand and address their individual struggles. Participants picture gloomy experiences of being abandoned and forced to navigate through life on their own. IPS awakens the possibility of obtaining the concrete help they need wherever required. The ES role blossoms into something greater, transcending the confines of job support to touch upon all facets of life: “A lot of things in my life changed. She has, as I said, helped me with things that are maybe not exactly within her … what is it called? Job description?” (P3).

From the stories of participants, ESs emerge as a provider of stability, offering practical support that ranges from securing housing, extended financial advice, ensuring mobility, or, in some cases, taking over as care coordinator. This support can be illustrated by one participant relating how the simple intervention as being driven to the university had a positive impact on her everyday life:

Just such a simple thing about this with the exam that I had when I was so afraid of not making it to the bus because the anxiety was so great (…). She drove me all the way, then it was out of the picture, then I could only focus on the actual exam and not the way there. And just getting one problem out of the way somehow makes things so much better. (P6)

Extended practical support that ESs provide can be further illustrated by the story of another participant who had a mental health assistant that needed to be urgently and unexpectedly replaced. The municipal mental health service argued that the participant had already established a strong relationship with the ES, and it would therefore be both legitimate and beneficial to be followed up by the ES at the same time, as the ES was still performing responsibilities within IPS. The participant agreed with this decision: “It was natural that she [ES] became the person I could lean on for more than just work, which ended up with her having two roles at the same time” (P6).

Most participants face severe financial problems, and this economic burden is drowning them in anxiety and distress, deteriorating their quality of life. This overwhelming situation is often exacerbated by the complexity, lack of transparency, and limited accessibility of the services that address financial issues. An ES is mentioned as the one providing financial counseling and reaching out to social services:

After a while she said, “take all the bills with you and we will discuss how to get everything fixed,” and she contacted the Norwegian State Educational Loan Fund, and she contacted NAV. Instead of me contacting NAV. NAV is a huge organization; I really don’t know how I was supposed to get around it on my own. (P1)

Participants clearly signalize the urgent need to address their needs, concerns, and challenges as well as express uncertainty about who has the authority to guide them. One participant voices not knowing where to address her long-term physical ailments: “I was thinking that they [mental health services] could help more with guiding you where you could get support and help with things. If you struggle with a bad spine, how should you move forward to get help?” (P2). ESs are able to see participants’ challenges and act on them quickly and efficiently to address their needs. In some cases, ESs take on an active role in care coordination, helping participants access various services to help them to get better and foster their recovery: “[ES] helped my therapist get in touch with the municipal mental health assistant for me” (P1). Another participant describes challenges with gaining weight that resulted in health problems and underlines the crucial role of ES in addressing those personal needs and managing difficulties: “Physically, I have put on 40 kg, and I feel that I have a lot of pain in my knees, neck, and back (…). But now I’m going to see a physiotherapist; The ES has arranged that” (P8).

Insufficient coordination between mental healthcare and other health services clearly emerges from participants’ accounts signalizing the urgent need to address their unmet needs. An ES appears as a vital asset in the lives of participants, being a messenger and a bridge between participants and the welfare system.

## Discussion

The aim of this study was to develop a deeper understanding of the IPS phenomenon as experienced by participants in everyday life. The essential meaning of the studied phenomenon has four constituents: (1) Having a safety net along the way toward employment; (2) Feeling more like a person, not just a patient; (3) Making the future look brighter; and (4) Going above and beyond employment support. While the first three constituents describe in depth the relationship between participants and ESs, the fourth constituent draws attention to the organizational context of IPS.

### Helping Relationship Between ES and IPS Participants

Addressing constituents 1–3, our study explores relational aspects between ESs and IPS participants. Many people experiencing mental illness struggle with various demographic, cognitive, clinical, psychosocial, and environmental barriers. Our study clearly illustrates that in those demanding conditions, engaging in job search might become possible only in safe surroundings when a trusting, personalized, and supportive relationship with an ES is established, and the continuity of the relationship is secured. Results from this study confirm existing findings about tailored and person-centered support, described by many IPS participants as the most relevant feature of the IPS model (Boycott et al., 2015; Topor & Ljungberg, 2016). The supportive relationships between ESs and IPS participants can be related to traditions within therapeutic alliance, also called working alliance (McCabe & Priebe, 2004). The key factors of this alliance are often described as the collaborative nature of this relationship, the trusting bond between patients and therapists and their ability to agree on treatment goals (Horvath & Symonds, 1991; Martin et al., 2000). In vocational support, the links between working alliance of ESs and IPS participants and job acquisition have been explored, with inconclusive results (Catty et al., 2008; Corbiere et al., 2024; Kukla & Bond, 2009). While this study doesn’t provide data on the relation between employment outcomes and the quality of relationships between ESs and IPS participants, it clearly illustrates that the continuity of stable support offered by ESs enables the creation of safe surroundings and trust, a “safety net,” essential to engage in the job search. Participants provide some detailed descriptions of ESs being receptive to their needs and able to create strong and engaging relationships based on trust and being valued, evoking a sense of safety.

While the relationship between ESs and IPS participants in this study fulfills criteria of working alliance based on collaboration and trust, it adds more to it. Those additional components support to create helping relationships. One element is the person-to-person investment approach, based not only on being seen and acknowledged but also on being allowed to be “ill and well at the same time” (Borg & Kristiansen, 2004, p. 496). Participants provide detailed descriptions of ESs treating them as fellow human beings and valued citizens instead of being reduced to symptoms and diagnosis. Participants in this study describe the challenges involved in engaging with public sector professionals. There are similar accounts in the literature of IPS participants’ experiences with professionals in conventional services failing to create resonating relationships (Rahbæk Møller & Bonfils, 2024) or labeling those relationships as “(…) impersonal and unproductive” (Gammelgaard et al., 2017, p. 402). Empowering collaboration with an ES described by IPS participants in this study departs from typical traditional clinician–client relationships. As a result, ESs manages to engage and motivate participants in the direction of occupational engagement.

The other vital component in helping relationships is creating hope. This study provides increased understanding of how, with help of a steady and reliable companionship of ESs, hope is being reawakened and sustained. Our participants describe experiencing hopelessness in everyday life, hindering them from engaging in everyday life activities. Being part of IPS enables them to experience hope and faith that positive changes are possible. The ES is an important part of this process, successfully performing the role of “holders of hope” (Glover, 2009, p. 71). Positive attitude, encouragement, and believing in one’s abilities are mentioned. This corresponds with findings from Areberg et al. (2013), wherein participation in IPS is described as “being the center of attention in a process that brings hope and meaning” (p. 591). Teixeira et al. (2020) described the “capacity to promote hope” as one of the core components of professional competencies needed to promote better vocational outcomes (p. 446).

### IPS in Relation to Health and Social Services

Relational aspects between ES’ and IPS participants in this study meet the principles of personal recovery understood as a personal, individually tailored path to development, a journey, not outcome. Personal recovery accentuates working towards improved mental health, regardless of diagnosis and symptoms. This contrasts with the paradigm of clinical recovery that emphasizes symptomatology and risk prevention (Slade, 2010). At the same time, in this study, ESs seem to depart from a personal recovery approach toward a more contextually oriented, relational recovery. Perspectives and experiences of personal recovery is an important challenge to the traditional biomedical approach to mental health. However, it is criticized for being individualistic (Price-Robertson et al., 2017) and “floating in a social vacuum” (Topor et al., 2022). Social determinants of mental health are repeatedly defined as fundamental determinant in mental health outcomes along the life span (Kirkbride et al., 2024). Relational recovery is founded on the idea of people being interdependent, functioning in social contexts. It recognizes interpersonal relationships as embedding all aspects of recovery, moving away from conceptualizing hope, empowerment, and identity as entirely intra-psychic processes (Price-Robertson et al., 2017).

Contextual factors do not just reflect the quality of the relationship between ESs and IPS participants but also seem to impact the range of tasks and responsibilities ESs fulfill. While studies associate ESs making flexible adjustments with IPS participants’ improved prospects of obtaining and holding a job (Kinn et al., 2021), our study described in constituent 4 provides various examples of how ESs clearly expand their vocational role. Some studies present examples of situations where ESs respond directly to non-vocational needs of IPS participants that seem to hamper the employment process. This includes loss of relatives, family conflicts, help with transportation, housing and medical insurance (Blitz & Mechanic, 2006; Gammelgaard et al., 2017), or even ESs providing their own clothes to IPS participants for a job interview (Whitley et al., 2010).

Given that ESs are often described as flexible, and adapting support to personal needs (Kinn et al., 2021), the present results clearly show that ESs are largely expanding their vocational responsibilities, addressing the gap in tailored support for persons experiencing SMI provided by several social services, including mental health services. According to Areberg et al. (2013), IPS participants report being abandoned by health and care providers who, in their opinion, offered little to no support at all. This study confirms those results and shows that IPS participants perceive the help provided by numerous health and social services as insufficient. On the contrary, ESs are responsive to the individual needs of IPS participants and address unmet needs and challenges of everyday life that are typically the domain of other public services.

Although participants in our study seem to benefit from ESs, getting help they need and are longing for, examples of diluting their role by regularly engaging ESs in non-vocational activities and practices raise questions around evidence-based principles of IPS and Supported Employment Fidelity Scale evaluations. This scale is used to assess the implementation of critical ingredients of IPS by determining the degree to which a specific program meets the standards of a model program (Bond et al., 2000, 2012). Higher fidelity scores are directly linked to higher success rates of the IPS model and good outcomes for individuals (Bond et al., 2012), while improvement of fidelity is correlated with improvement of employment outcomes over time (de Winter et al., 2020). One of the 25 items of the scale clearly states that ESs have an exclusive vocational focus and do not provide non-vocational services (Bond et al., 2012). At the same time, “The presence of a full time ES’ who provided only vocational services was significantly correlated with higher rates of competitive employment” (Kirsh et al., 2005, p. 273). There is, therefore, a risk that, due to ESs involvement in non-vocational activities, fewer IPS participants succeed in gaining and retaining employment.

This study raises questions about how much more than just vocational services ESs actually provide in practical settings and how are the outcomes of IPS and the everyday life of IPS participants affected. Additionally, it is crucial to reflect over the reasons for ESs systematically and broadly expanding their role, engaging in non-vocational services, while IPS is integrated and provided in parallel with mental health treatment.

Our results illustrate that IPS participants experience the support from care services as fragmented, not properly coordinated, and difficult to access. Many mental health service users agree that support provided by different actors is not coordinated; there is a lack of continuity within and across organizations hampering cooperation and bureaucratic structures, which makes it difficult to establish and maintain contact with different services (Germundsson et al., 2011; Nygren et al., 2016). Coordination at the organizational level plays a crucial role in the impact of IPS by providing resources and structures for professionals to work (Sundermann et al., 2023). Therefore, coordinated efforts from several agencies must work successfully to enable persons experiencing SMI to obtain employment. For example, turnover of ESs is significantly higher than the average turnover rate of other occupations in the public sector (Butenko et al., 2022), owing to implementation challenges such as integration of ESs into healthcare services and the lack of clarity of the delegation of roles and responsibilities among sectors (Butenko et al., 2022; Moe et al., 2021). Despite ongoing attempts at promoting system integration among service providers and users (World Health Organization, 2022), mental health and social services both in Scandinavian countries (Borg & Kristiansen, 2008; Germundsson et al., 2011) and internationally (Xyrichis & Lowton, 2008) continue to be fragmented and lack inter-professional, cross-disciplinary, and inter-organizational cooperation. This often leaves the burden of coordinating help on individuals and their families (Borg & Kristiansen, 2008; Wirsén et al., 2020).

Healthcare systems appear to not only be fragmented and struggle with coordination but are also highly hierarchical, with hierarchical cultures, operating a top-down structure of management. The delivery of healthcare services has therefore to be considered through the impact of professional and social hierarchies (Essex et al., 2023). The healthcare organizations face challenges related to authority and power in patient care, and there is increasing pressure for inclusion of collaborative models of care, flattering the institutional hierarchy and redistributing power (Noyes, 2022; WHO, 2022). Although IPS is intended to be integrated into mental health treatment and the access to the program is granted due to diagnostic criteria, ESs provide support in non-health settings and are receptive to everyday life issues of IPS participants. Our research shows that applied in practice, an ES has the autonomy to act independently and provide holistic and personalized help that health services seem not to fully cover.

### Strengths and Limitations

Studies on IPS lack the involvement of persons with lived experiences in the research processes (Rahbæk Møller et al., 2020). Involving peer researchers in this study holds the potential to make the study more responsive to users’ needs (Veseth et al., 2013) and enables better contextualization, communication, and application of the findings (Devotta et al., 2016). Regarding limitations, several IPS participants declined to participate in this study. The main reason for this was, as reported by ESs, a high level of social anxiety. This may have limited variation of the experiences shared, especially in terms of potentially negative experiences with IPS among non-participants. Finally, the study was carried out in Norway, and contextual variations in the organization and functioning of mental health services, as well as diversity in implementation strategies of the IPS model, could influence the understanding of IPS as a phenomenon in different settings.

## Conclusions

This study adds in-depth and contextual knowledge to the growing research on participants’ experiences of IPS and illustrates ESs moving from traditional oriented personal recovery toward more contextually oriented relational recovery. This means in practice that ESs provide individualized assistance in supporting persons in living a meaningful life based on their choices, strengths, and abilities, shifting attention from diagnosis and challenges into creating possibilities. This relationship is a source of strength for IPS participants and enables them to regain hope for the future and improve self-esteem, in contrast to relationships with mental health professionals that participants describe as often lacking personalized support and having too much focus on illness and risk management. This individualized assistance is contextually oriented, growing from and acknowledging the role and impact of everyday life of IPS participants not only on their vocational outcomes but also on quality of life. The participants experience support and follow-up received from health and care services as insufficient and describe these services are fragmented, uncoordinated, and difficult to access. ESs, not being a part of hierarchical medical services, tend to act independently, providing holistically and relationally oriented approach addressing a broad range of participants’ unmet needs. This challenges the fidelity criteria of IPS. Mental health services that provide more contextually oriented care and better coordination between vocational and health services hold potential to positively affect people’s vocational outcomes and quality of life.

Further qualitative studies on IPS participants’ experiences in the organizational and societal contexts, which could be helpful for further development of the IPS model, are warranted. In addition, in both the international and Norwegian contexts, the experiences of family members and friends of IPS participants (Keefe et al., 2020) are left to explore. Moreover, there is an urgent need to include persons with lived experiences in the research process to contribute to the democratic development of knowledge in mental healthcare, especially in the everyday life context.
